# Schizophrenia risk ZNF804A interacts with its associated proteins to modulate dendritic morphology and synaptic development

**DOI:** 10.1186/s13041-021-00729-2

**Published:** 2021-01-14

**Authors:** Fengping Dong, Joseph Mao, Miranda Chen, Joy Yoon, Yingwei Mao

**Affiliations:** Department of Biology, Pennsylvania State University, 214 Life Sciences Building, University Park, PA 16802 USA

**Keywords:** Schizophrenia, ZNF804A, ZNF804A binding proteins, Dendritic morphology

## Abstract

Schizophrenia (SZ) is a devastating brain disease that affects about 1% of world population. Among the top genetic associations, *zinc finger protein 804A* (*ZNF804A*) gene encodes a zinc finger protein, associated with SZ and biolar disorder (BD). Copy number variants (CNVs) of *ZNF804A* have been observed in patients with autism spectrum disorders (ASDs), anxiety disorder, and BD, suggesting that *ZNF804A* is a dosage sensitive gene for brain development. However, its molecular functions have not been fully determined. Our previous interactomic study revealed that ZNF804A interacts with multiple proteins to control protein translation and neural development. ZNF804A is localized in the cytoplasm and neurites in the human cortex and is expressed in various types of neurons, including pyramidal, dopaminergic, GABAergic, and Purkinje neurons in mouse brain. To further examine the effect of gene dosage of *ZNF804A* on neurite morphology, both knockdown and overexpression of *ZNF804A* in primary neuronal cells significantly attenuate dendritic complex and spine formation. To determine the factors mediating these phenotypes, interestingly, three binding proteins of ZNF804A, galectin 1 (LGALS1), fasciculation and elongation protein zeta 1 (FEZ1) and ribosomal protein SA (RPSA), show different effects on reversing the deficits. LGALS1 and FEZ1 stimulate neurite outgrowth at basal level but RPSA shows no effect. Intriguingly, LGALS1 but not FEZ1, reverses the neurite outgrowth deficits induced by ZNF804A knockdown. However, FEZ1 and RPSA but not LGALS1, can ameliorate ZNF804A overexpression-mediated dendritic abnormalities. Thus, our results uncover a critical post-mitotic role of ZNF804A in neurite and synaptic development relevant to neurodevelopmental pathologies.

## Introduction

In 2008, O’Donovan and colleagues identified the *ZNF804A* as the first gene to reach the genome-wide significance associated with SZ [1]. The rs1344706 in the second intron of *ZNF804A* shows a strong association with both SZ and BD. The associations between *ZNF804A* and SZ were also confirmed by other groups [2–4]. In 2014, SZ Working Group of the Psychiatric Genomics Consortium compared 36,989 schizophrenia cases with 113,075 controls and identified 108 conservatively genomic loci that reached genome-wide significance [5]. *ZNF804A* is still among the one of top candidates. Besides single nucleotide polymorphisms (SNPs), CNVs of *ZNF804A* have been reported in patients with psychiatric diseases [3]. *ZNF804A* CNVs, including duplication and deletion, were also observed in patients with ASDs [6], supporting that *ZNF804A* risk is not only limited to SZ. These results also suggest that *ZNF804A* dosage is critical and altered expression may lead to various developmental disorders.

*ZNF804A* is highly expressed in brain tissues. The expression of *ZNF804A* in the brain increases during the early fetal stage and reaches the peak at mid-fatal stage (13–24 post-conceptual weeks), and decreases after birth [7]. We have also demonstrated that mouse ZFP804A, an orthologue of human ZNF804, is highly expressed in the embryonic mouse brain and is decreased in adulthood [8]. The human-induced pluripotent stem cells (iPSC) from SZ patients expressed *ZNF804A* at the early differentiation stage [9]. *ZNF804A* knockdown in cultured human neural progenitors (NPs) affects the expression of genes regulating cell adhesion, mitosis, neural differentiation, and inflammatory response [10]. ZNF804A regulates genes that are involved in transforming growth factor β (TGFβ) signaling pathway [11]. The targets of ZNF804A include several SZ risk genes, including catechol-O-methyltransferase (COMT), phosphodiesterase 4B (PDE4B) and dopamine receptor D2 (DRD2) [12]. These results indicate that the gene-dosage of ZNF804A affects the transcriptome and cellular function.

ZNF804A is localized at neurites and dendritic spines [13]. Downregulation of *Zfp804a*, a rodent orthologue of human *ZNF804A*, significantly reduces the neurite outgrowth and the density of dendritic spines in rat primary cortical neurons, suggesting an important role of ZNF804A on neurite outgrowth and maturation. We have demonstrated that *Zfp804a* is highly expressed in the embryonic mouse brain and decreased expression in adulthood [8]. ZNF804A physically interacts with different proteins of SZ risk genes and developmental genes to regulate neurogenesis. However, how ZNF804A interacts with its binding partners to modulate neuritogenesis is unclear. This study tests how gene dosage of *ZNF804A* affects neurite outgrowth and spine development. We show that both upregulation and downregulation of *ZNF804A* could significantly impair dendritic morphology. Intriguingly, several ZNF804A binding proteins show distinct capacities on reversing the dendritic and synaptic defects. Collectively, our results demonstrate that ZNF804A plays a critical role in the neuronal development and interacts with multiple factors to modulate to these cellular processes, which are relevant to the progression of psychiatric diseases.

## Results

### ZNF804A protein in mouse and human brain

**Fig. 1:**
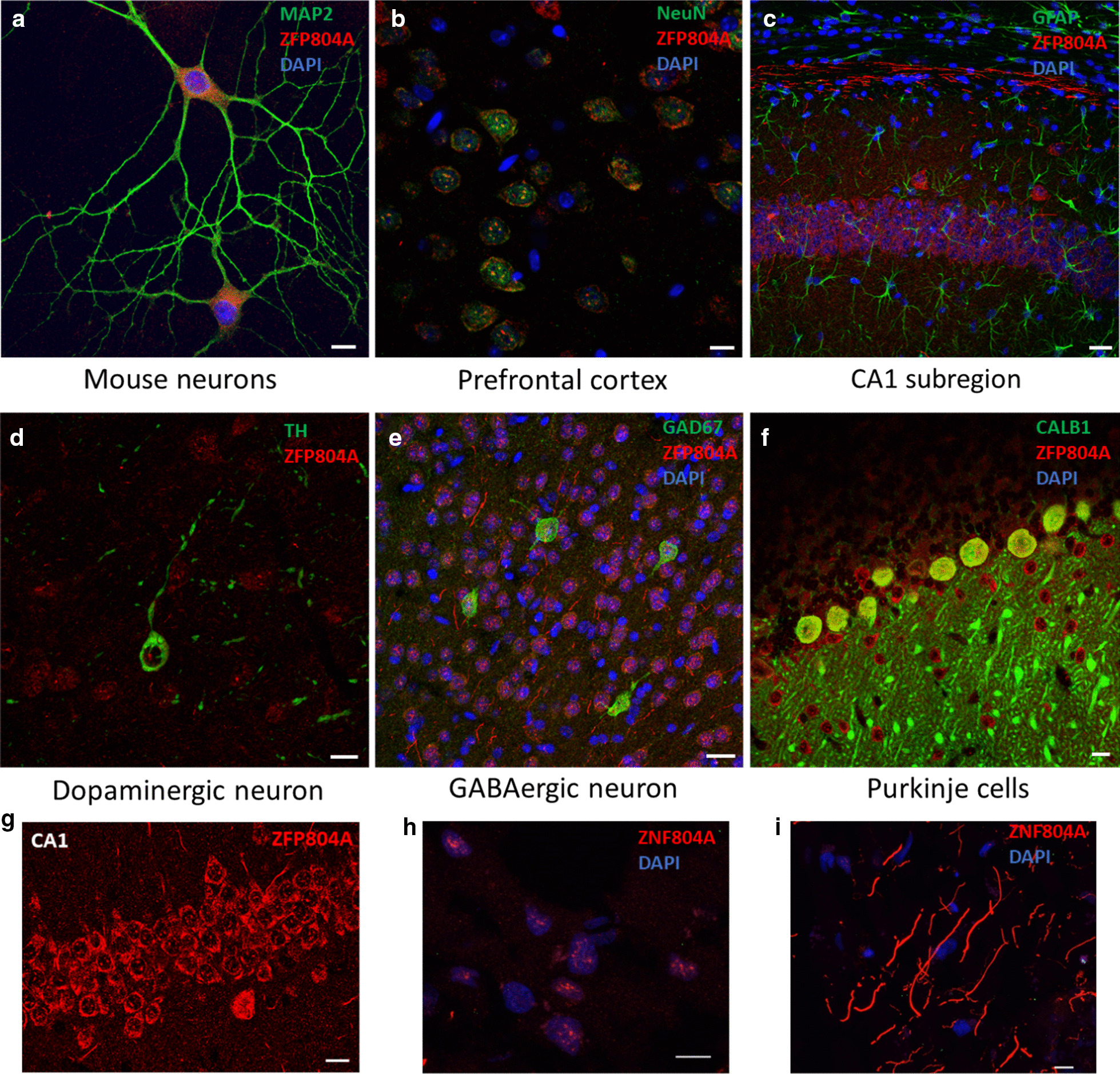
ZNF804A antibody immunostaining demonstrated the localization of ZFP804A in primary cultured mouse neurons (**a**). ZFP804A is highly expressed in the nuclear as well as in cytoplasm and neurites. In the mouse brain, ZFP804A is exclusively expressed in neurons (**b**) with no detectable signal in astrocytes (**c**). ZFP804A expressed in different types of neurons, for example dopaminergic neurons (**d**), GABAergic neurons (**e**), and Purkinje cells (**f**). ZFP804A is highly expressed in neurites (arrows) (**g**). Bar = 10 µm. **h**, **i** Immunostaining demonstrates that ZNF804A is highly expressed in nucleus of human neurons (**h**) and neurites (**i**) at the cingulate cortex. Bar = 10 µm

In the human brain, the expression of *ZNF804A* mRNA increases from the embryonic to the early fetal stage and reaches a peak around the early-mid fetal stage. It decreases afterward and stays at a constant level until late adulthood [7]. As mRNA may not correlate with protein level, we first examined ZNF804A protein distribution in the brain. Consistent with our previous study, we observed the ZFP804A expression in primary cultured mouse neurons (Fig. 1a). In the adult mouse brain, ZFP804A is highly expressed in neurons (Fig. 1b) not in astrocytes (Fig. 1c). Multiple types of neurons showed ZFP804A signal, including dopaminergic neurons (Fig. 1d), GABAergic neurons (Fig. 1e), and Purkinje neurons (Fig. 1f). The immunostaining results confirmed that ZFP804A is localized in the nucleus, cytoplasm, and neurites of CA1 neurons (Fig. 1g). Consistently, ZNF804A is localized in both nucleus and cytoplasma of neural cells in human brain sections of the cingulate cortex (Fig. 1h). Intriguingly, ZNF804A is highly enriched in some dendrites in the cingulate cortex (Fig. 1i), suggesting a potential role of ZNF804A in neurite development.

### ZNF804A knockdown affects the neurite outgrowth and spine formation

**Fig. 2:**
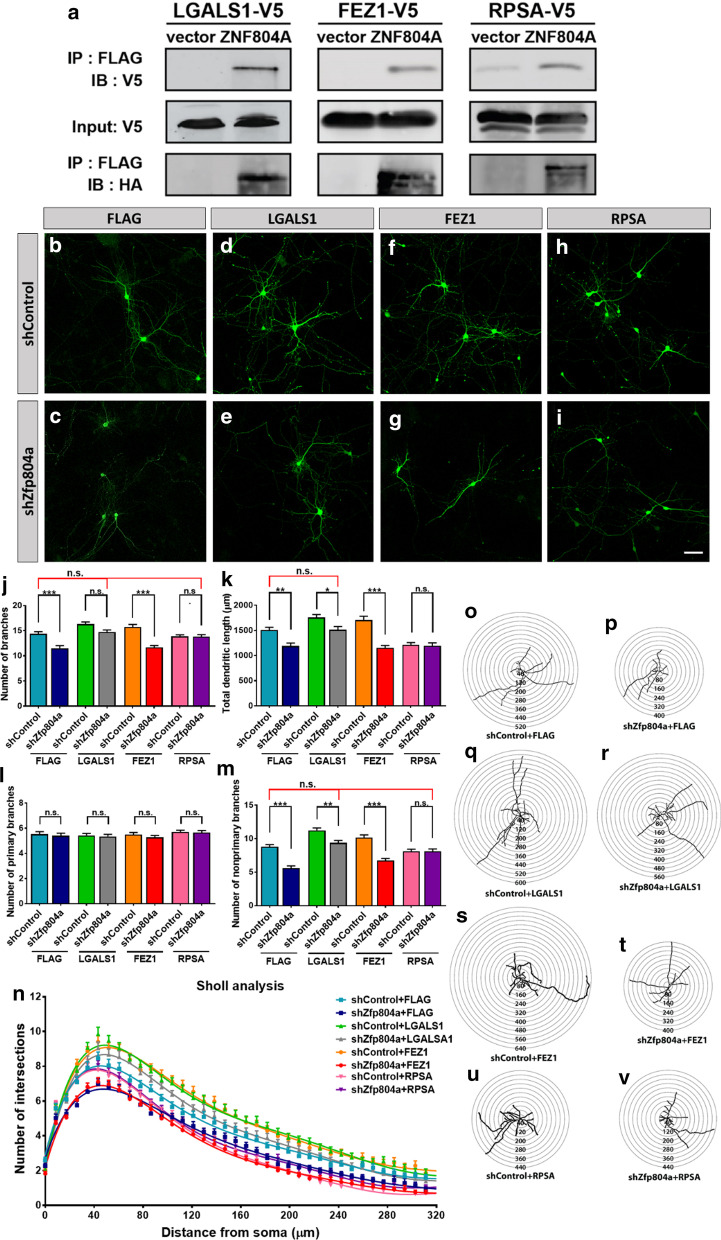
**a** ZNF804A protein physically interacts with LGALS1, FEZ1, and RPSA. (**b**, **i**) *Zfp804a* shRNA or a scramble shRNA are co-transfected with constructs expressing FLAG tag (**b**, **c**), LGALS1 (**d**, **e**), FEZ1 (**f**, **g**) or RPSA (**h**, **i**) separately into primary mouse cortical neurons. The transfected neurons are stained with GFP (green). Bar = 50 µm. *Zfp804a* knockdown in differentiating neurons affects number of branches (**j**), total dendritic length (**k**), number of primary branches (**l**), and number of non-primary branches (**m**). The Sholl analysis indicates the complexity of the neurite outgrowth (**n**), and illustrates representative co-transfected neurons with concentric circles (**o**–**v**). The interval between adjacent consecutive circles is 10 μm (n = 70)

ZNF804A protein is enriched in neurites (Fig. 1a, g, i) and we have identified a group of ZNF804A interacting proteins involved in the neurite outgrowth using yeast-2-hybrid system (Y2H) [8] including FEZ1 and LGALS1. FEZ1 interacts with microtubules to enhance the extension of neurites [14, 15]. *LGALS1* encodes galectin-1 that normally promotes neurite outgrowth and axonal regeneration [16, 17]. The neuronal migration deficits could be rescued by overexpression of the ZNF804A-interacting protein RPSA [8], indicating that protein–protein interactions could provide potential therapeutic targets for restoring deficits caused by genetic abnormalities. We hypothesize that these interactions can shape the neurite development. To test this hypothesis, we first confirmed the physical interactions of ZNF804A with 3 proteins, LGALS1, FEZ1 and RPSA, by co-immunoprecipitation (Fig. 2a).

We next tested the effect of ZNF804A interacting proteins on *Zfp804a-*mediated neurite outgrowth. We co-transfected a FLAG tag vector or plasmids expressing LGALS1, FEZ1 and RPSA with a shRNA construct that we have shown the efficient knockdown of *Zfp804a* [8] into primary mouse cortical neurons (Fig. 2b–i). We further confirmed the efficacy of silencing in mouse N2a cells (Additional file 1: Figure S1). The effects on dendritic morphology were analyzed in Fig. 2j–m. *Zfp804a* shRNA significantly suppressed total number of neurite (*P* = 0.0239) (Fig. 2j), total dendritic length (*P* = 0.0325) (Fig. 2k), and the number of non-primary neurites (*P* = 0.0027) (Fig. 2m), but not the number of primary neurite (Fig. 2l). Sholl analysis confirmed the defect of neurite outgrowth caused by *Zfp804a* knockdown (Fig. 2n–p).

Intriguingly, LGALS1, rather than FEZ1, rescued the neurite deficits caused by *Zfp804a* knockdown (Fig. 2). LGALS1 recovered total number of neurite branch (*P* = 0.5843), dendritic length (*P* = 0.9999), and number of non-primary neurite (*P* = 0.408) (Fig. 2j–m). The Sholl analysis indicated that LGALS1 increased the number of intersections between neurites and the consecutive circles in the *Zfp804a* downregulation neurons (Fig. 2n, q, r). FEZ1 failed to rescue the neurite outgrowth deficits, and showed similar pattern as *Zfp804a* knockdown (Fig. 2j–n, s, t). RPSA restored total number of neurites (*P* = 0.9981) and non-primary neurites (*P* = 0.7505), but not the dendritic length (*P* = 0.0001) compared to shControl + FLAG condition (Fig. 2j–m). The Sholl analysis showed similar complexity of neurite outgrowth in RPSA overexpression group regardless *Zpf804a* levels (Fig. 2n, u, v). Multiple comparisons of knockdown experiments were summarized in Additional file 1: Table S1.

**Fig. 3:**
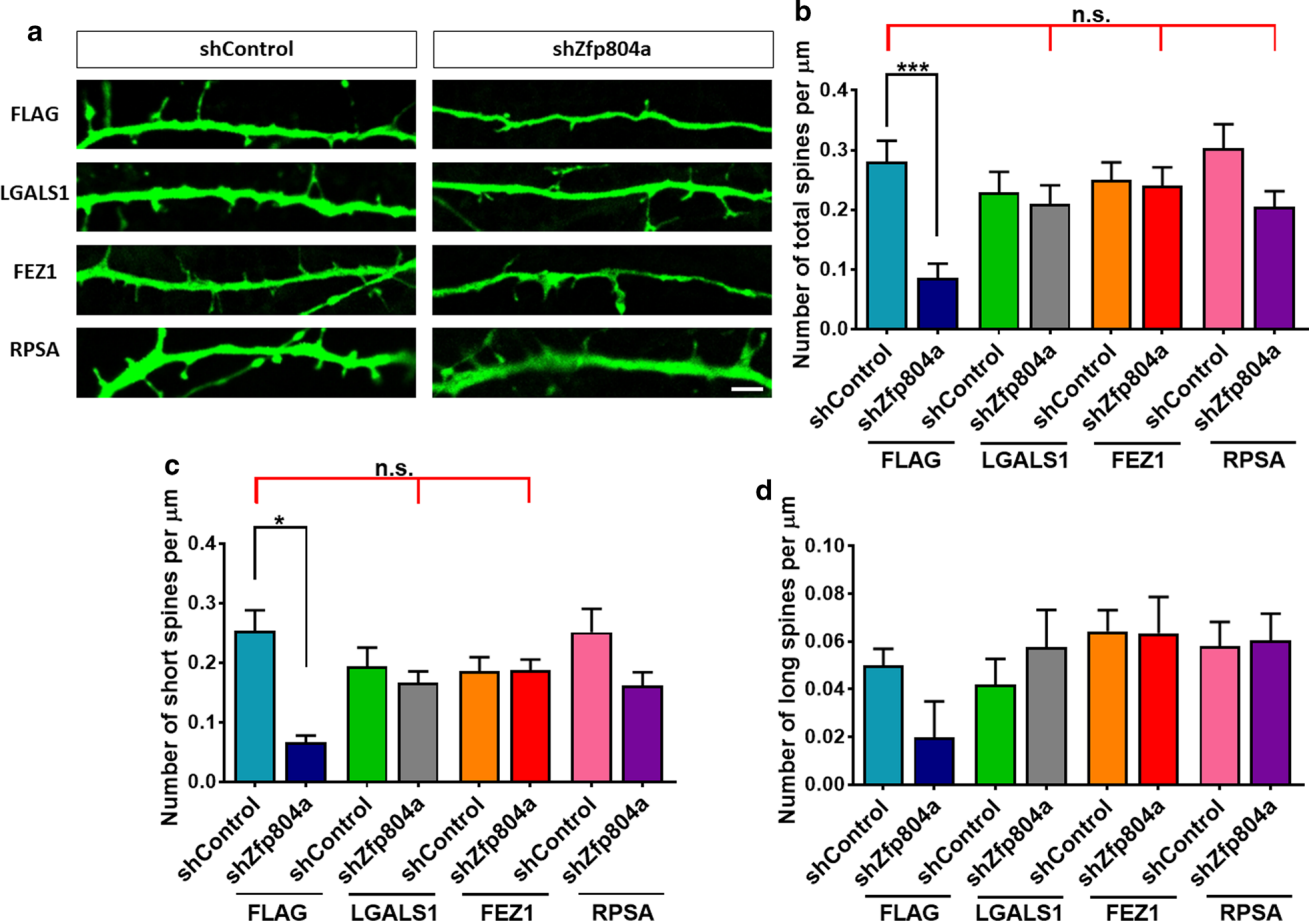
The spine density of transient co-transfect mouse neurons was measured (**a**) *Zfp804a* knockdown significantly reduces spine formation (**b**–**d**), LGALS1, FEZ1, and RPSA reverse the reduction of total spine number caused by *Zfp804a* downregulation (**b**). LGALS1 and FEZ1 restore short spines as well as long/thin spines (**c**, **d**). Bar = 2 µm

Dendritic spines are essential for receiving inputs from synapses. The density and plasticity of dendritic spines play a fundamental role in neural connections. The morphological development of dendritic spines reflects their maturity [18]. A dendritic filopodia with a long neck and small head are considered to be an immature spine. It becomes shorter and reach to its maturity with a morphology of a mushroom head and a short neck [19]. The spine formation was significantly reduced by *Zpf804a* shRNA (Fig. 3a). Downregulation of *Zfp804a* decreased the total spine density (*P* = 0.0003) and short spines (*P* < 0.0001) (Fig. 3b–c), but not long spine number (Fig. 3d). LGALS1, FEZ1, or RPSA overexpression at basal level (with control shRNA) did not affect total dendritic spine density (Fig. 3a, b). Interestingly, LGALS1, FEZ1, or RPSA ameliorated the defect of total spine density (Fig. 3b), whereas LGALS1 and FEZ1 reversed the defect of short spine density comparing to the control group (Fig. 3c). These data suggest that ZNF804A interacting proteins play different roles in ZNF804A-mediated dendritic/spine development.

### ZNF804A overexpression reduces the neurite outgrowth and spine density

**Fig. 4:**
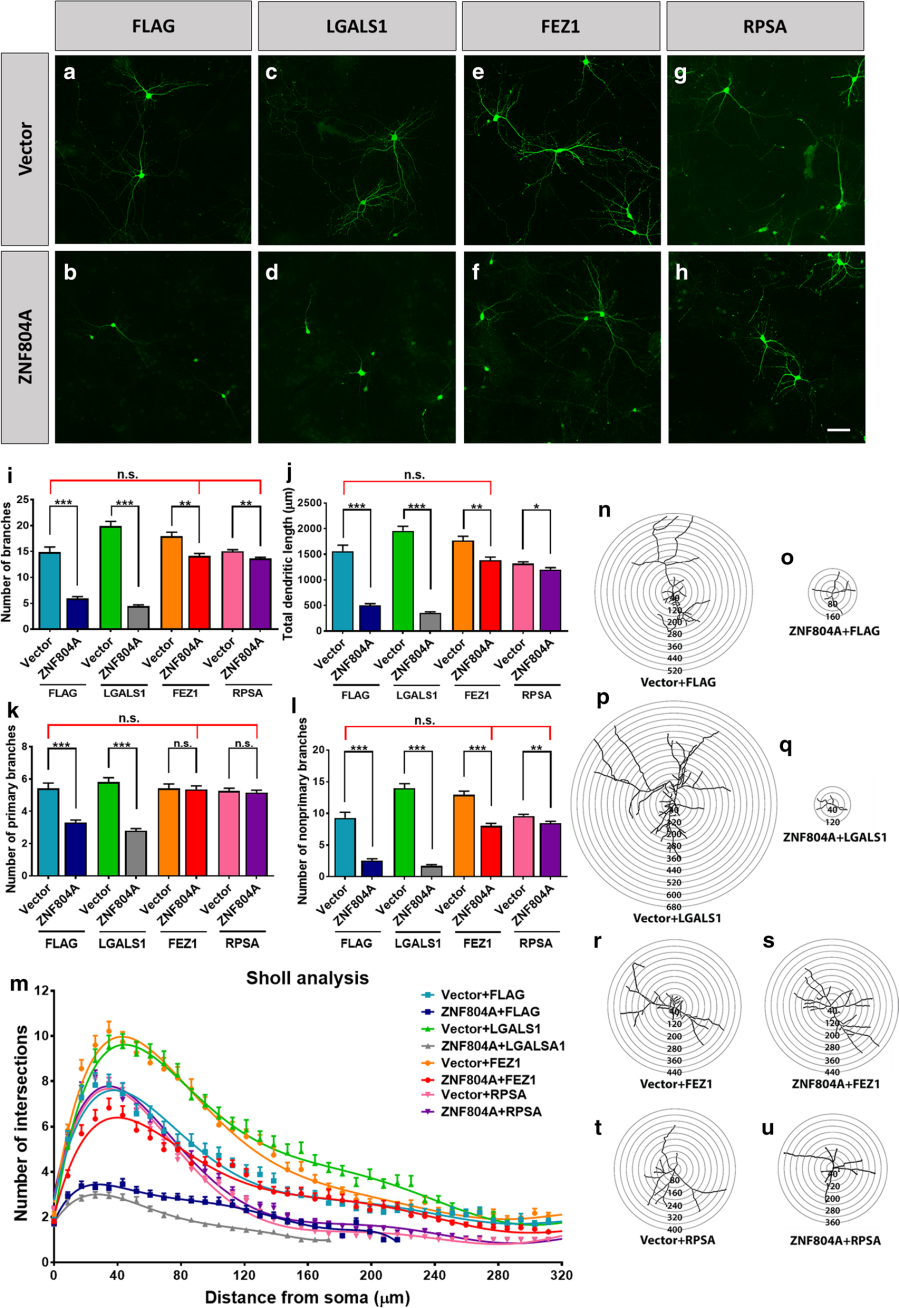
ZNF804A and empty GFP overexpression vectors were co-transfected with vectors expressing FLAG tag (**a**, **b**), LGALS1 (**c**, **d**), FEZ1 (**e**, **f**) or RPSA (**g**, **h**) separately into primary neurons. The transfected neurons were stained with GFP (green). Bar = 50 µm. ZNF804A overexpression in differentiating neurons leads to reduced number of branches (**i**) and total dendritic length (**j**), number of primary branches (**k**), and number of non-primary branches (**l**). FEZ1 restored deficits of both neurite number and neurite length. FEZ1 and RPSA increase the primary (**k**) and non-primary (**l**) neurite number that are decreased by ZNF804A overexpression. The Sholl analysis shows the complexity of the neurite outgrowth (**m**), and illustrates representative co-transfected neurons with concentric circles (**n**–**u**). The interval between adjacent consecutive circles is 10 μm (n = 70)

We previously demonstrated that overexpression of ZNF804A enhances translation rate [8] and duplication of ZNF804A is reported in patients with psychiatric illnesses [6]. To determine the biological function of a high level of ZNF804A in neurons, we co-transfected ZNF804A construct with plasmids expressing LGALS1, FEZ1, or RPSA into primary cortical neurons (Fig. 4a–h). Surprisingly, we observed a much stronger phenotype in neurite outgrowth in ZNF804A overexpressing neurons (Fig. 4a, b).

Overexpression of ZNF804A significantly reduced the number of dendritic branches (*P* < 0.0001) and total dendritic length of neurons (*P* < 0.0001) (Fig. 4i, j). The number of both primary and non-primary neurites were decreased (Fig. 4k, l). The Sholl analysis support a significantly reduced number of neurite branches (Fig. 4m–o).

LGALS1 promoted overall neurite outgrowth in the control group, significantly increasing the number of dendritic branches (Fig. 4i) and dendritic length (Fig. 4j). Specifically, LGALS1 overexpression significantly increased the number of non-primary neurites (Fig. 4l), rather than primary neurites (Fig. 4k). However, LGALS1 overexpression failed to restore aberrant neurite outgrowth in the ZNF804A overexpressing group (Fig. 4i–l). The Sholl analysis confirms the insufficiency of LGALS1 in recovering ZNF804A overexpression-medicated dendritic defects (Fig. 4m, p, q).

FEZ1 is known to directly bind to DISC1 (*disrupted in schizophrenia 1*) protein, an important SZ risk, to regulate neurite outgrowth [14]. Notably, we found that FEZ1 increases the dendritic length and the number of branches in ZNF804A overexpression group (Fig. 4i, j). In vector-expressing cells, FEZ1 stimulates outgrowth in non-primary neurites similar as LGALS1 (Fig. 4k, l). In contrast to LGALS1, FEZ1 overexpression reversed deficits of primary and non-primary neurites induced by ZNF804A overexpression (Fig. 4k, l). Further Sholl analysis showed that FEZ1 overexpression restored the number of intersections between neurites and the proximal consecutive circles (Fig. 4m, r, s). This evidence indicated that FEZ1 rescues neurite outgrowth deficits caused by ZNF804A overexpression.

Interestingly, RPSA overexpression recovers the total number of neurites as well as both primary and non-primary neurites (Fig. 4I, K-L). However, RPSA alone shows a trend to reduce the total dendritic length (P = 0.695), and cannot rescue the deficits caused by ZNF804A overexpression (P = 0.0007) (Fig. 4j). The Sholl analysis confirmed our observation that the intersections between neurites and the consecutive circles are not reduced until 80 µm circle (Fig. 4m, t, u).

**Fig. 5:**
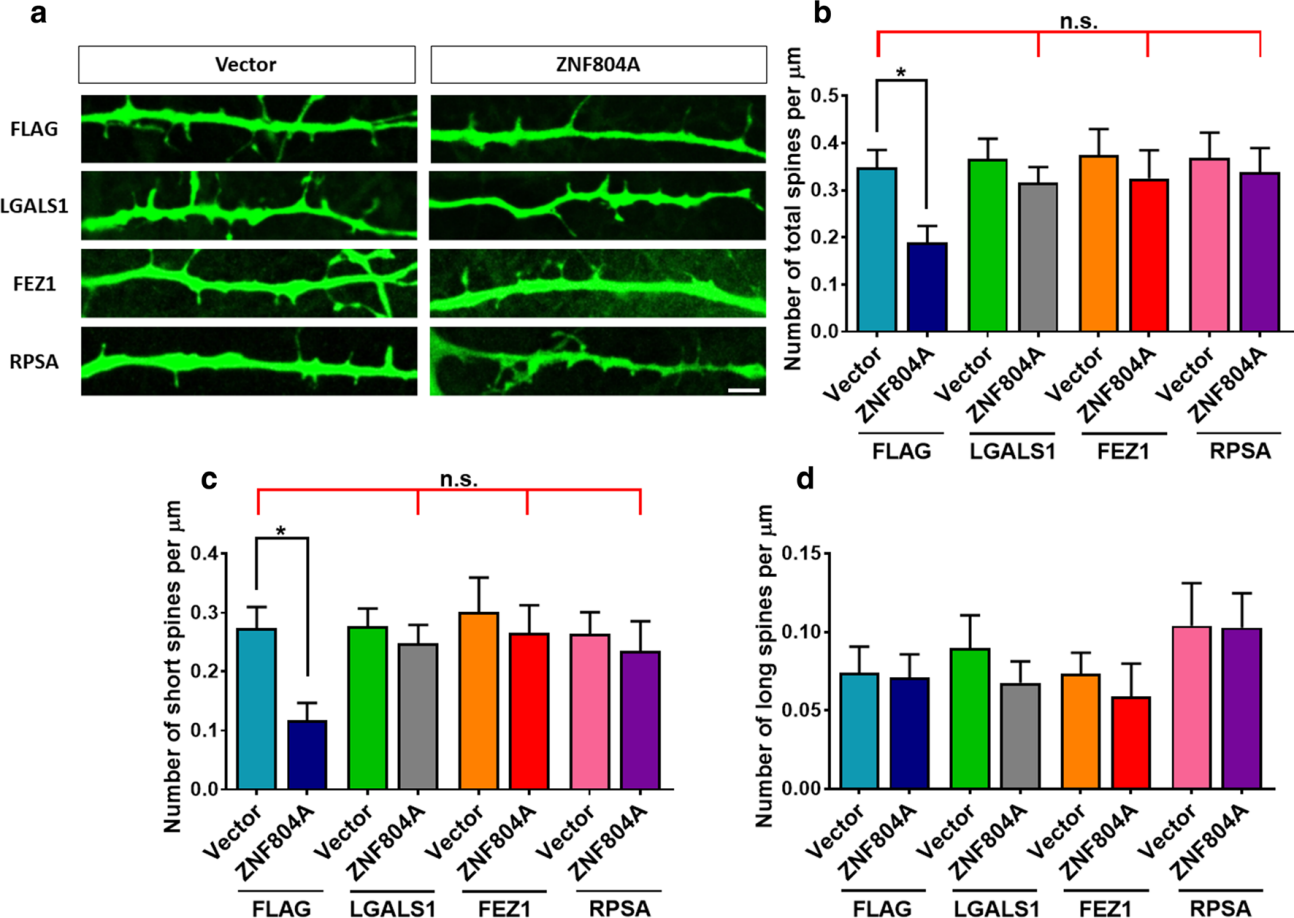
The spine density of transient co-transfect mouse neurons was measured (**a**). ZNF804A overexpression significantly reduces total spine formation (**b**) and the number of short spines (**c**), but not long/thin spines (**d**). LGALS1, FEZ1, and RPSA alleviate the deficits of spine formation caused by ZNF804A overexpression (**b**, **c**). Bar = 2 µm

We next analyzed the spine density and morphology in the primary cultured mouse neurons with ZNF804A overexpression. The dendritic spines density showed a significant reduction in the ZNF804A overexpressed mouse neurons (P = 0.0328) (Fig. 5a, b). Further analyzing spine morphology indicated that the short spines (P = 0.0229) are decreased significantly (Fig. 5c) but the long/thin spine density is intact (Fig. 5d). Surprisingly, LGALS1, FEZ1, and RPSA could recover the deficits of reduced spine density caused by ZNF804A overexpression (Fig. 5a, b). They showed similar number of short spines with a slightly elevated number of long/thin spine only in the RPSA overexpression group (Fig. 5c, d). Multiple comparisons of overexpression experiments were summarized in Additional file 1: Table S2.

## Discussion

ZNF804A is highly expressed in NPs and reaches a peak at E14 in the embryonic mouse brain [8]. ZFP804A is expressed in neurons but not in astrocytes (Fig. 1), suggesting ZNF804A plays an essential role in neurodevelopment. Here we demonstrated that in addition to modulating NP differentiation and neuronal migration, both upregulation and downregulation of ZNF804A significantly impair dendritic and synaptic spine development. Intriguingly, ZNF804A upregulation produces a much severer neurite phenotype. Moreover, we showed that ZNF804A interacting proteins, LGALS1, FEZ1 and RPSA, exhibit different abilities to alleviate the defects caused by abnormal ZNF804A levels. These data support that an optimal level of ZNF804A is required for normal dendritic morphology and suggest that ZNF804A interacts with different binding partners to modulate differentiation processes.

Studies have shown that neurite outgrowth deficits have been associated with dysfunctional SZ risk genes, such as *neuregulin 1* (*NRG1*), *DISC1*, *AKT serine/threonine kinase 1* (*AKT1*), and *dystrobrevin binding protein 1* (*DTNBP1*) [14, 20, 21]. Interestingly, both knockdown and overexpression of *ZNF804A* affect the expression of genes related to cell adhesion [10, 22]. Consistently, our Y2H results also identify that ZNF804A interacts with multiple cell adhesion proteins [8], suggesting that neurite defects could be associated with adhesion deficits caused by ZNF804A deregulation. Downregulation of *Zfp804a* represses neuronal migration to the cortical plate in the mouse embryonic state [8]. Neurite outgrowth deficits were observed in the primary cultured mouse neuron with *Zfp804a* knockdown. The number of primary neurites, which originate from the soma, is unaffected by reducing *Zfp804a*. The number of non-primary neurites as well as short total neurite length is significantly reduced. Consistently, knockdown of *ZNF804A* attenuates neurite outgrowth in young human iPSC-derived neurons [13].

Psychiatric disorders often share associated risk loci with each other. A meta-analysis of eight mental disorders identified 109 risk SNP loci associated with at least two disorders. Many ASD risk loci are also associated with SZ and BD [23]. Besides SNPs, CNVs, such as chromosomal deletion or duplication, have been implicated in psychiatric disorders. Both microdeletion and microduplication of 1q21.1 and 17p12 are associated with SZ and ASD [24]. CNV also happens within a single gene. Complex CNVs of *contactin associated protein 2* (*CNTNAP2)* gene associated with several diseases across different phenotypes [25]. Interestingly, Steinberg and colleagues identified two *ZNF804A* deletions from a Scottish patient with SZ and an Icelandic patient with anxiety, and one *ZNF804A* duplication in an Icelandic patient with BD [3]. ZNF804A overexpression significantly attenuates neurite outgrowth and dendritic spine formation. The reduction of the primary neurite generation is a distinct deficit between the overexpression and knockdown of *ZNF804A*. As *ZNF804A* overexpression increases mRNA translation [8], our study reveals the complex mechanisms regulating neurite outgrowth by *ZNF804A*.

Interacting proteins can reveal the potential molecular and biological functions of a protein. More importantly, protein–protein interactions could serve as targets to rescue the disease phenotypes caused by risk genes. For example, the MDM2-p53 interaction became a valuable target for developing cancer therapy [26]. The interaction proteins of ZNF804A were identified by the Y2H experiment in our previous study [8]. Two major functional groups, translation, and cell adhesion were clustered with multiple genes. LGALS1 and FEZ1 were reported with the capability of regulating neurite outgrowth [16, 17]. RPSA has a dual function as an RNA-binding protein and a laminin receptor to control cell adhesion [27]. Intriguingly, these interacting proteins exhibit different recovery potential against either overexpressed or downregulated ZNF804A. Both LGALS1 and FEZ1 stimulated overall neurite outgrowth. Only FEZ1 rescues the neurite outgrowth deficits caused by *ZNF804A* overexpression, whereas only LGALS1 recovers deficits induced by *Zfp804a* knockdown.

*LGALS1* encodes protein galectin-1, a modulator of cell adhesion [28], which is localized in the nucleus, cytoplasm and extracellular compartments binding to beta-galactoside [17]. It also plays an important role in neuroprotection by regulating microglia activation in the central nervous system [29, 30]. Interestingly, galectin-1 is found significantly higher in the unaffected siblings of SZ patients compared to both the patient group and the healthy control group [31]. Consistent with previous studies that LGALS1 promotes neurite outgrowth and axonal regeneration [32, 33], our study shows that LGALS1 stimulates non-primary neurite outgrowth. LGALS1 rescues the neurite deficits caused by *Zfp804a* downregulation, whereas FEZ1 does not. *Zfp804a* knockdown generates a mild defect compared to overexpression (Figs. 2, 4). The ability to override deficits induced by *Zfp804a* knockdown suggests that LGALS1 could target shared downstream genes or pathways of *Zfp804a.* Interestingly, it activates FAK/PI3K/AKT/mTOR Pathway [34, 35] that is critical for neurite growth and brain development [36]. Consistently, we have shown that ZNF804A can also modulate PI3K/AKT/mTOR Pathway [8]. Thus, a possible mechanism of LGALS1 to reverse *Zfp804a*-mediated deficits may go through activation of FAK/PI3K/AKT/mTOR Pathway, which could be tested in the future studies.

FEZ1 is known to physically interact with DISC1 [37], a notable risk linked to SZ [14]. DISC1 plays a critical role in neurodevelopment, and its mutations lead to deficits in cell adhesion and neurite outgrowth [14, 38]. It regulates cell proliferation, differentiation, and migration through GSK3β/β-catenin pathway [39]. Dopamine D2 receptors affect neurites via dopamine D2 receptor‐DISC1‐GSK3β signaling [40]. Either hyper dopamine D2 receptor activation by over release of dopamine or blocked dopamine D2 receptors by its antagonist will lead to neurite outgrowth deficits [40]. ZNF804A physically interacts with FEZ1 to recover attenuated neurite outgrowth, indicating that FEZ1 is required for neurite elongation and fasciculation in mammals [41]. Intriguingly, both FEZ1 [42] and DISC1 [43] can modulate PI3K/AKT pathway. Thus, in addition to physical interaction, PI3K/AKT/mTOR Pathway could be a convergent pathway regulated by ZNF804A, FEZ1, LGALS1 and DISC1. FEZ1, ZNF804A, LGALS1 and DISC1 may act at various steps upstream of the PI3K/AKT/mTOR pathway to control neurite growth. These complicate interactions will in turn fine-tune PI3K/AKT/mTOR pathway through activation or inhibition.

*RPSA*, encodes as a p40 ribosome-associated protein and a laminin receptor (37/67-kDa laminin receptor/ LAMR), involves in diverse biological functions. RPSA has been implicated in neurodegenerative diseases and developmental aberrations [44]. RPSA knockdown attenuates neurite outgrowth [45]. We previously demonstrated that RPSA rescues neuronal migration deficits caused by knockdown of *Zfp804a* in the mouse embryonic state [8]. Overexpression of RPSA also decreases the high level of cytoplasmic translation observed in neurons. Interestingly, RPSA overexpression recovers the neuron morphology by increasing the neurite number in both ZNF804A overexpression and *Zfp804a* knockdown group. However, it also inhibits the total dendritic length. Neurites with RPSA overexpression may take a longer time to reach the same length as controls. Alternatively, lacking sufficient laminin may reduce the in vitro neurite elongation. Other genes, such as NLGN4X, also reverse deficits of neurite length against the downregulation of ZNF804A in human neurons [13]. In summary, future therapeutic approaches of psychiatric diseases should be beneficial from careful studies of the critical protein–protein interactions.

## Materials and methods

### Animal and human postmortem samples

All procedures on mice were reviewed and approved by the Pennsylvania State University institutional animal care and use committee (IACUC), under IACUC protocol number 44057–1. Wild type male and female C57BL/6N mice were obtained from Taconinc. Mice were hosted by sex (2–5 mice per cage) in a room with a light/dark cycle at 12 hr intervals, and provided ad libitum access to food and water. Frozen human postmortem brain sections (14 µm) of the cingulate cortex from normal control subjects were provided by Stanley Medical Institute.

### Neuronal culture and transfection

Primary cortical neurons were cultured as described [46]. Briefly, the embryonic cortex (embryonic day 14) was isolated in iced Hank's Balanced Salt Solution (HBSS) and digested with 0.25% trypsin with DNase I for 5 min at 37 °C. After adding trypsin inhibitor, digested tissues were pipetted to break up into single cells. Cells were spun down at 1000 revolutions per minute (RPM) at 4 °C for 5 min. Spun cells were washed with HBSS and suspended with Dulbecco's Modified Eagle Medium (DMEM)/F-12 with 1% Penn-Strep, 1% L-glutamine, 1X N-2 and B27 supplements. Cells were plated into a 24-well plate with Poly-D-lysine pre-coated coverslips. Culture media were replaced with fresh media every other day.

Primary neurons were transient transfected with plasmids at day in vitro (DIV) 2 to overexpress *ZNF804A*, or to knock down *Zfp804a* with shRNA (5′-CAGAGAGAATTTGCTCG AAATG-3′). An empty vector for overexpression or a shRNA vector with scramble sequence (5′-GGCTCCCGTGAATTGGAATCC-3′) served as the negative control [8]. ZNF804A interacting partners, FEZ1, LGALS1, and RPSA, were co-transfected. A sham vector expressing FLAG tag were used as the negative control. The Calcium phosphate transfection was performed as described in Additional file 1 [47].

### Immunostaining

Cultured cells were grown in on coverslips until mature. To fix cell samples, the culture medium was removed and washed with phosphate-buffered saline (PBS) for two times. Following the fixation with 4% paraformaldehyde for 10 min at room temperature, cells were then washed with PBS twice. Cells were then blocked with 5% donkey serum in PBS with 0.1% Triton X-100 for 1 h. The primary antibodies were mixed in blocking buffer with appropriate dilution factors. After blocking, cells were incubated with primary antibodies overnight. Coverslips were washed with 0.3% Triton X-100 in PBS for three times. Cells were then incubated with Alexa secondary antibodies that conjugated with fluorescein (Invitrogen). After additional washing with PBS, cells were mounted with ProLong Gold antifade Reagents (Life Technologies).

### Sholl analysis

Immunostaining images were scanned by Carl Zeiss LSM 5 Pa confocal microscope. We analyzed the neurite number, length, and complexity of 40–60 individual neurons in each group by using the Sholl Analysis plugin integrated in Fiji [48]. The neurite derived from the soma of a neuron was considered as a primary neurite, and the neurite derived from a primary neurite was considered as a nonprimary neurite.

### Dendritic spine analysis

To analyze the dendritic spine, a z-stack image was used to include all visible spines. Dendritic spines were classified as short (< 2 µm) or long/thin (> 2 µm) based on their length. Spine density was calculated as the number spine per µm.

### Statistical analysis

Data were analyzed using Excel and SPSS software and are expressed as means ± standard error of the mean (SEM). Significances between the experimental groups and control groups were analyzed by two-way ANOVA and multiple comparison test. The threshold of significance was set to *P < 0.05; **P < 0.01; ***P < 0.001.

## Supplementary Information

**Additional file 1.**. Additional figure and tables.

## Data Availability

The reagents are available from the corresponding author upon request.

## Abbreviations

SZ: Schizophrenia
BD: Bipolar disorder
CNVs: Copy number variants
ASDs: Autism spectrum disorders
ZNF804A: Zinc finger protein 804A
Zfp804a: Zinc finger protein 804A
LGALS1: Galectin 1
FEZ1: Fasciculation and elongation protein zeta 1
RPSA: Ribosomal protein SA
GABA: Gamma-aminobutyric acid
iPSCs: Induced pluripotent stem cells
SNP: Single nucleotide polymorphism
NPs: Neural progenitors
TGFβ: Transforming growth factor β
COMT: Catechol-O-methyltransferase
PDE4B: Phosphodiesterase 4B
DRD2: Dopamine receptor D2
DIV: Day in vitro
shRNA: Small hairpin RNA
NRG1: Neuregulin 1
DISC1: Disrupted in schizophrenia 1
AKT1: AKT serine/threonine kinase 1
DTNBP1: Dystrobrevin binding protein 1
CNTNAP2: Contactin associated protein 2
IACUC: Institutional animal care and use committees
RPM: Revolutions per minute
SEM: Standard error of the mean
ANOVA: Analysis of variance
HBSS: Hank’s balanced salt solution
DMEM: Dulbecco’s modified Eagle m*ed*ium
PBS: Phosphate-buffered saline
Y2H: Yeast-2-hybrid
