# Supplementary material for: Schizophrenia risk ZNF804A interacts with its associated proteins to modulate dendritic morphology and synaptic development
Source: Mol Brain. 2021 Jan 14;14:12. doi: 10.1186/s13041-021-00729-2 (PMC7809827; doi:10.1186/s13041-021-00729-2)
Supplement: Supplementary file 1 — Additional file 1.. Additional figure and tables. [file 13041_2021_729_MOESM1_ESM.docx]

**Schizophrenia risk ZNF804A interacts with its associated proteins to modulate dendritic morphology and synaptic development**

Fengping Dong, Joseph Mao, Miranda Chen, Joy Yoon, Yingwei Mao^1,*^

**Additional Materials and Methods**

**Cell culture and transfection**

Human Embryonic Kidney 293tsA1609neo (HEK293T) cells were cultured in DMEM – high glucose (Sigma) with 10% fetal bovine serum (Atlanta Biologicals), 10 unit/mL penicillin and 10 µg/mL streptomycin (Gibco). The culture medium was replenished every two days, and cells were sub-cultured when reaching 80% to 90% confluency.

Transfection was performed as described [1], human HEK293T cells or mouse N2a neuroblastoma cells were plated in six-well plates at 3 × 10^5^ cells per well or in 10 cm dishes at 3 × 10^6^ cells. After reaching 70% of confluency, cells were transfected with polyethyleneimine (PEI)- plasmids complex. Briefly, cells were fed with fresh culture medium one hour before transfection. Plasmid DNA and PEI were diluted into individual tubes with a weight ratio of 1:3. The sequences for shRNAs targeting mouse Zfp804a are as follows: shControl: 5′-GGCTCCCGTGAATTGGAATCC-3′ against firefly luciferase; shZfp804a: 5′-CAGAGAGAATTTGCTCGAAATG-3′; shZfp804a 2: 5′-TCCTTTGCATTTCCAAAGAAAG-3′ as described in reference [2].The PEI solution was mixed with DNA solution and incubated for 15 min at room temperature to form a complex. The PEI-plasmid complex solution was dropwise into culture dishes. Transfected cells were incubated for overnight in a CO_2_ incubator and cultured for an additional 48 hours with fresh culture medium.

**Co-immunoprecipitation and Western blot**

ZNF804A overexpression constructs LV-EF1α-hZNF804A-3xFLAG-3xHA-P2A-EGFP and its control LV-EF1α-3xFLAG-3xHA-P2A-EGFP were separately transfected into HEK293T cells with N-terminal V5-tagged FEZ1, LGALS1, and RPSA using polyethyleneimine (PEI) transfection approach [3]. Transfected cells were changed with fresh cell culture medium after 12 hours, and collected in PBS on ice after 72 h. Cell pellet were lysed with ice-cold RIPA buffer, containing 50 mM Tris HCl (pH7.5), 150 mM NaCl, 1.0% (v/v) NP-40, 0.5% Sodium Deoxycholate, 1.0 mM EDTA, 0.1% SDS and 0.01% sodium azide with complete protease inhibitor cocktail (Sigma) added. After incubated on ice for 15 min, samples were centrifuged for 20 min at 12000 rpm at 4 C. Supernatants were transferred into a new tube. The concentration of protein was measured by the Bradford method [4]. A small aliquot of the lysate was denatured at 100°C for 10 min with 6x Laemmli loading buffer, consisting of 0.375 M Tris pH 6.8, 12% SDS, 60% glycerol, 0.6 M DTT, and 0.06% bromophenol blue.

Lysates with 2 mg protein were transferred into a tube with 25 µl ANTI-FLAG^@^ M2 affinity gel (Sigma). After overnight incubation with rotation at 4 C, beads were spun down, and supernatants were aspirated off. To remove the non-specific binding, beads were washed with lysis buffer four times, and PBS with 0.05% Triton X-100 twice. After the last wash, beads were boiled within 30 µl of 2x Laemmili loading buffer to release the binding protein.

An equal amount of protein as inputs and denatured pull-down products from experimental and control groups were loaded to an acrylamide gel with an appropriate percentage of acrylamide. Protein was separated with electrophoresis at 70 V for 20 min and 120 V for 70 min and transferred onto nitrocellulose membranes. Immunoblots were performed as described (Mao & Lee, 2005). The membranes were blocked in TBS (10 mM Tris–HCl, pH 8.0, 150 mM NaCl) with 5% non-fat milk for 1 h at room temperature and blotted with primary antibodies in the blocking buffer for overnight. The secondary antibodies, donkey anti-mouse IgG-800 and anti-rabbit IgG-700 (LI-COR), were used to blot with membranes for 1 h at room temperature. Images were acquired and analyzed through Image Studio^TM^ according to the instruction of Odyssey CLx Infrared Imaging System.

**Neuronal transfection**

Briefly, in a sterile microcentrifuge tube, 1 µg plasmid DNA, 3.1 µL 2M CaCl_2_ were mixed with sterile H_2_O to the total volume of 25 µL. In another microcentrifuge tube, the same volume of 2 x HBS was prepared. In order to reach the high efficiency of transfection, the DNA and CaCl_2_ mixture was dropwise added into the tube with 2 x HBS with a gentle vortex. The mixture was incubated for 5 min at room temperature and dropwise added to the coverslip with primary cortical neural progenitors. After 30 min incubation in 5% CO_2_ incubator at 37°C, the coverslip was incubated for 15 min with a medium that had been pre-equilibrated with 10% CO_2_. The coverslip was transferred back to the 24-well plate containing the original culture medium.

**Immunohistochemistry**

To obtain brain samples for immunostaining, mice were perfused with PBS until all blood is removed and perfused with 4% PFA to pre-fix tissues. The mouse brain was dissected carefully with tweezers and post-fixed with 4% PFA at 4°C overnight. Then, the fixed brain was transferred into PBS to remove PFA residue. Brain slices with the thickness of 40 µm were obtained by vibratome (Leica) and treated with pepsin/HCl cleaning solution (Mettler Toledo) for 10 min. Brain slices were then blocked with blocking buffer (PBS with 0.3% Triton X-100 and 5% donkey serum) for 1 h. Primary antibodies were diluted in the blocking buffer and incubate with brain slices overnight with gentle shaking. The next day, brain slices were washed three times with PBS with 0.05% Triton –X100 and incubated with fluorescein-labeled secondary antibodies for 2 h. Brain slices were mounted to glass slides with Prolong Gold Antifade (Invitrogen). Immunostaining steps were carried out at room temperature.


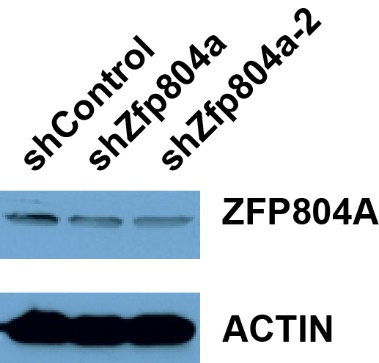


**Additional Figure S1.** **Confirmation of ZFP804A knockdown.** Western blot analysis of the efficacy of ZFP804A knockdown shRNA. Two shRNAs-shZfp804a and shZfp804a-2 efficiently knocked down endogenous ZNF804A in mouse N2a cells.

**
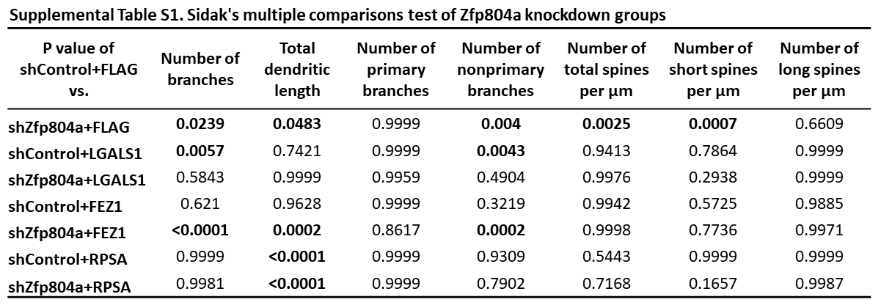
**

**
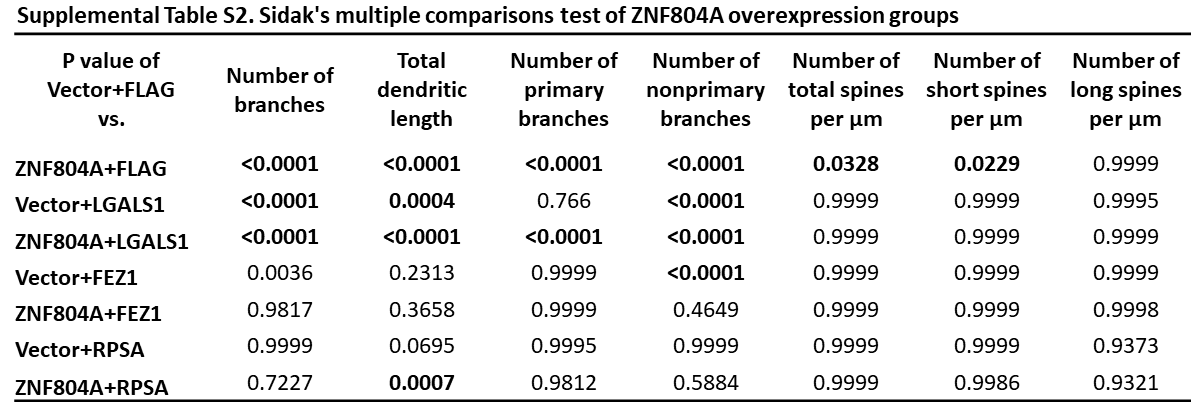
**

**References**

1. Goncalves C, Gross F, Guegan P, Cheradame H, Midou P: **A robust transfection reagent for the transfection of CHO and HEK293 cells and production of recombinant proteins and lentiviral particles - PTG1.** *Biotechnol J* 2014, **9:**1380-1388.

2. Zhou Y, Dong F, Lanz TA, Reinhart V, Li M, Liu L, Zou J, Xi HS, Mao Y: **Interactome analysis reveals ZNF804A, a schizophrenia risk gene, as a novel component of protein translational machinery critical for embryonic neurodevelopment.** *Mol Psychiatry* 2018, **23:**952-962.

3. Longo PA, Kavran JM, Kim MS, Leahy DJ: **Transient mammalian cell transfection with polyethylenimine (PEI).** *Methods Enzymol* 2013, **529:**227-240.

4. Kruger NJ: **The Bradford method for protein quantitation.** *Methods Mol Biol* 1994, **32:**9-15.
